# Understanding stroke and spasticity: patient and caregiver experiences and quality-of-life impact

**DOI:** 10.3389/fneur.2026.1849865

**Published:** 2026-10-15

**Authors:** Monica Verduzco-Gutierrez, Emilie Pain, Jonathan Bouchard, Amandeep Mann

**Affiliations:** 1 UT Health San Antonio, San Antonio, TX, United States; 2 Carenity, Online Patient Community, Paris, France; 3 Ipsen, Cambridge, MA, United States

**Keywords:** cross-sectional survey, online, quality-of-life, spasticity, stroke

## Abstract

**Introduction:**

Poststroke spasticity (PSS) is a common sensorimotor disorder following a stroke, presenting in up to 38% of patients 1 year after first stroke. Owing to its heterogeneous and progressive presentation, PSS represents a significant challenge both in its effect on patients and in its clinical management. The objective of this US real-world survey was to assess the impact of self-reported PSS symptoms, gathering patient and caregiver perspectives on awareness of PSS and its treatments, the extent of shared decision-making, and the quality of life (QoL) impact of PSS.

**Methods:**

This study was a noninterventional, cross-sectional, web-based survey of patients (or their caregivers) within 1 year of stroke resulting in hemiparesis. Participant demographics and survey responses were summarized using descriptive analyses. Subgroup analyses were performed between patients and caregivers and by sex, age group, and type of last stroke. A short version of the Stroke-Specific QoL (SS-QoL) scale was used to assess patient QoL.

**Results:**

The study included 221 patients and 62 caregivers answering on behalf of patients. Almost all patients (98%) experienced PSS symptoms after their most recent stroke, with 59% experiencing ≥3 symptoms. Approximately half (56%) of patients who reported PSS symptoms were aware of drug treatments; of these, most (95%) had received ≥1 drug treatment. Among treated patients, 68% had been involved in the initial drug selection. Over half of patients reported difficulties carrying out daily activities, but caregivers were more likely to report difficulty showering or bathing unassisted (84%) compared with the patient group (58%); fatigue was reported by most patients (84%). The overall median (IQR) SS-QoL score was 2.5 (2.0–2.9) and was higher in the patient group (2.5; 2.1–2.9) than the caregiver-reported group (2.1; 1.8–2.8). Our real-world survey findings demonstrate a considerable impact of PSS on patient QoL, largely driven by fatigue and limitations in daily activities. Furthermore, subgroup analysis revealed significant differences between patient and caregiver perceptions of PSS symptoms and their impact.

**Discussion:**

This study highlights the need for increased awareness of PSS among healthcare providers, patients, and caregivers to promote timely diagnosis and improve management with targeted, multidisciplinary interventions that enhance patient outcomes and support caregivers.

## Plain language summary

After a stroke, some people experience a condition called poststroke spasticity (PSS) that causes muscle stiffness and uncontrolled spasms. Symptoms are not the same for everyone with PSS and may get worse over time. This survey-based study described how people with PSS and their caregivers were affected by PSS after a stroke. The survey included questions related to a person’s knowledge about the condition, access to treatments, and how PSS impacts their daily life. The researchers also looked at differences in people’s responses based on their age, sex, and the type of stroke they experienced, as well as whether the patient or their caregiver completed the survey. Nearly all of the people who took the survey experienced PSS symptoms after their most recent stroke, but only half of them were receiving drug treatment for their PSS symptoms. People most often reported being affected by fatigue and difficulty in doing daily activities, such as showering or bathing. Also, caregivers reported a greater impact on quality of life than patients themselves. This study emphasized the need for education about PSS to improve diagnosis, management, and support for patients and their caregivers.

## Introduction

Stroke is a leading cause of long-term disability worldwide (1). Despite medical advances, a significant proportion of stroke survivors experience long-term disability, with motor impairments being reported in approximately 80% of cases (2). Poststroke spasticity (PSS), a common sensorimotor disorder to develop following a stroke, is a manifestation of upper motor neuron syndrome caused by central nervous system lesions, and arises from hyperexcitable descending excitatory brainstem pathways (3–6). PSS is characterized by a velocity- and muscle length-dependent increase in muscle tone with exaggerated resistance to externally imposed stretching of resting muscles (6). It manifests in approximately 1 in 4 survivors within 2 weeks following the initial stroke (7) with more cases emerging over time; approximately 38% of patients are affected 1 year after a first stroke and 44% after a recurrent stroke (6, 8, 9). Notably, younger stroke survivors are at higher risk for developing PSS (10, 11). The heterogeneous and progressive nature of PSS presents a significant challenge for clinical management; patients with PSS often require long-term care but frequently report limited access to treatment, highlighting an unmet need within the healthcare system (2, 8).

Delayed diagnosis, inadequate referrals to rehabilitation, and limited access to treatment hinder effective management, despite the existence of clinical guidelines emphasizing early intervention and prevention (12–14). The clinical manifestations of PSS range from intermittent, subtle neurologic manifestations to severe, often painful muscle tone increases, which can lead to joint immobility in approximately 15% of poststroke patients (2). Furthermore, paresis, defined as the partial loss of voluntary muscle function or muscle weakness, is reported in almost 40% of stroke survivors following a first stroke (15). The progression of PSS over time is driven by a vicious cycle in which persistent muscle overactivity leads to stiffness, contractures, and reduced mobility, further reinforcing maladaptive neural plasticity and exacerbating functional impairment (16).

If left untreated or inadequately managed, PSS can lead to worsening severity and reduced quality of life (QoL) for stroke survivors and also for their caregivers (12–14). Caregiver burden is reported to be substantial among those caring for patients with PSS, and is positively associated with the level of patient disability (17, 18). Caregivers exhibit poorer physical and emotional health than the general population (18). In addition, caregivers of patients with PSS face productivity loss and bear significant indirect costs associated with caregiving (19). More research is needed to understand specific contributors to this caregiver burden (17).

The economic burden of stroke care is significant, largely owing to prolonged hospital stays and the ongoing healthcare costs associated with risk management and long-term rehabilitation (20, 21). Furthermore, healthcare costs for stroke survivors with PSS are 2 to 4 times higher compared to those without PSS (22, 23). Beyond its clinical impact, the economic burden of spasticity underscores the urgent need for better preventive strategies, policy-driven interventions, and public health initiatives promoting healthier lifestyles (20). Nevertheless, the limited awareness and understanding of PSS, in conjunction with the absence of standardized multidisciplinary poststroke care, frequently hinder patients from accessing timely and cost-effective treatments (24, 25).

The objective of this observational survey was to gather real-world evidence on PSS collected from patients in current routine clinical management, as well as perspectives from their caregivers. The survey assessed the following: (i) patient awareness and education about the condition and available treatments, (ii) the extent of shared decision-making in treatment choices, and (iii) the impact of PSS on patient QoL. The acquisition of in-depth insight into the real-world experience of patients through self-reported data enhances the understanding of their unmet needs, expectations, and challenges in their clinical management. These findings may also guide policymakers and clinicians in prioritizing preventive measures and developing strategies to reduce the burden of PSS.

## Methods

### Study design

This noninterventional, cross-sectional web-based survey was conducted between July and November 2023. Patients and caregivers were recruited online through several channels. First, recruitment was conducted through Carenity, an online community for patients living with chronic conditions and their caregivers, where members can share experiences, provide support, access health-related information, and voluntarily contribute to medical research by participating in online studies. Registration on Carenity is free and open to adults (≥18 years of age). During registration, members provide basic demographic and health information, consent to the Terms of Use and the processing of their personal health data, and may opt in to receive invitations to participate in online research related to their condition. Members of the US Carenity community who have declared having experienced a stroke were invited to participate to the study through emails or private messages. To broaden recruitment beyond the Carenity community, the study was also disseminated through Carenity’s partner panels (specialized in online study recruitment) and through online advertising campaigns (Facebook and Google Advertisements).

Eligible participants were patients aged ≥18 years who declared they had suffered a stroke in the past year resulting in hemiparesis, or their caregivers. Respondents also had to be fluent in English and residing in the United States to be eligible for inclusion. For the purposes of this study, PSS was not clinically confirmed but was self-reported by participants according to the presence of at least one of the following symptoms potentially associated with poststroke spasticity: Muscle spasms, muscle stiffness/rigidity (including painful cramps), muscle pain, unwanted movement of the affected arm and/or leg, difficulties moving the leg, which may result in falling, tripping, loss of balance, difficulties moving the arm/hand, extending the arm, and/or opening the hand (6, 26)

The survey included a total of 23 questions on the sociodemographic and medical profile of the patient, their experience with stroke and spasticity, and the impact of stroke on the patient’s QoL (see Supplementary Appendix). Questions were identical for patients and caregivers answering on behalf of a patient. Patients who answered the survey on their own constituted the “patient group” while caregivers who completed the survey on behalf of the patients constituted the “caregiver group.” All participants provided electronic informed consent prior to completing the survey. The survey was designed and approved by a multidisciplinary steering committee. The study was reviewed and approved by the WCG independent institutional review board (IRB) prior to commencement (IRB tracking number: 20233447).

### Analysis

Descriptive analyses were conducted to characterize the study population and summarize responses to the survey. The data were presented as median and interquartile range (IQR), mean and SD, or frequency and percentage values as appropriate. Subgroup analyses were performed, notably based on whether the patient belonged to the “caregiver group” or the “patient group.” Additional subgroup analyses were conducted by sex, age groups (determined by the median), and the type of the last stroke experienced (ischemic or hemorrhagic).

To evaluate patients’ QoL, a short version of the Stroke-Specific Quality of Life Scale (SS-QoL) was used (27). This version of the SS-QoL included 12 items related to trouble performing daily tasks (taking a bath or shower, walking, buttoning buttons, repeating themselves after speaking, watching television, daily chores around the house) and other life aspects (trouble remembering things, feeling like a burden to the family, interference with social life, change in personality). Points were allocated to each respondent depending on the answers given for each item, and the overall SS-QoL score was computed as the unweighted average of all items. The overall score ranged between 1 and 5—a lower score indicating a more severely impacted QoL and a higher score indicating a better QoL.

A minimum target sample size of 130 patients or caregivers was predefined, with at least 30 participants per subgroup of interest (patient/caregiver, gender, age, type of the last stroke experienced) to address study objectives, ensure the collection of reliable descriptive data, allow for meaningful descriptive and exploratory subgroup analyses, and account for feasibility based on previous studies conducted by Carenity in the stroke population. Exploratory bivariate and subgroup analyses were used to assess differences in the responses reported by different subgroups of patients: patients vs. caregivers, age categories (above vs. below the median), sex, and types of last stroke, without adjustment for multiple comparisons. Student *t* tests or Wilcoxon’s rank sum tests were used to compare quantitative characteristics between these subgroups of patients, and chi-square and Fisher’s exact tests were used to compare categorical characteristics. All statistical tests were two-sided, and *p* values <0.05 were considered statistically significant. Multivariate linear regressions were used to model the relationship between the SS-QoL and several independent variables (profile, sex, age, types and number of comorbidities, awareness of drug treatment availability, types of therapy and treatment received, symptoms) in the subgroup of patients whose most recent stroke in the past 12 months was a first stroke event. Coefficients and predicted means were calculated and presented with their 95% CI and associated *p* value. A first multivariate linear regression model was performed and included all the factors associated with the SS-QoL in bivariate analyses at a *p* value <0.2. The adjustment variables were selected by a stepwise procedure, using the Akaike Information Criterion (AIC). The final model obtained was the one with the lowest AIC value including only factors that remained significantly and independently associated with the SS-QoL.

Data management and analysis were performed using R software (version 4.1.2; R Core Team).

## Results

### Participant and patient profiles

Of the 432 participants who completed the survey, 149 were removed owing to poor data quality (e.g., inconsistencies between answers, identical rating on all scales, too little time spent on the questionnaire) (Figure 1). The survey took approximately 12 min on average to complete. Among the 283 respondents included in the final study sample, 221 (78%) were patients and 62 (22%) were caregivers answering on behalf of patients. The median patient age was 48 years (IQR, 39.0–59.5), with a slightly greater proportion of patients ≥48 years of age (48–59 y, 27%; 60–91 y, 25%) compared with <48 years of age (21–39 y, 29%; 40–47 y, 19%). Men comprised 55% (n = 155) of the sample. Medical characteristics are detailed in Table 1.

**Figure 1:**
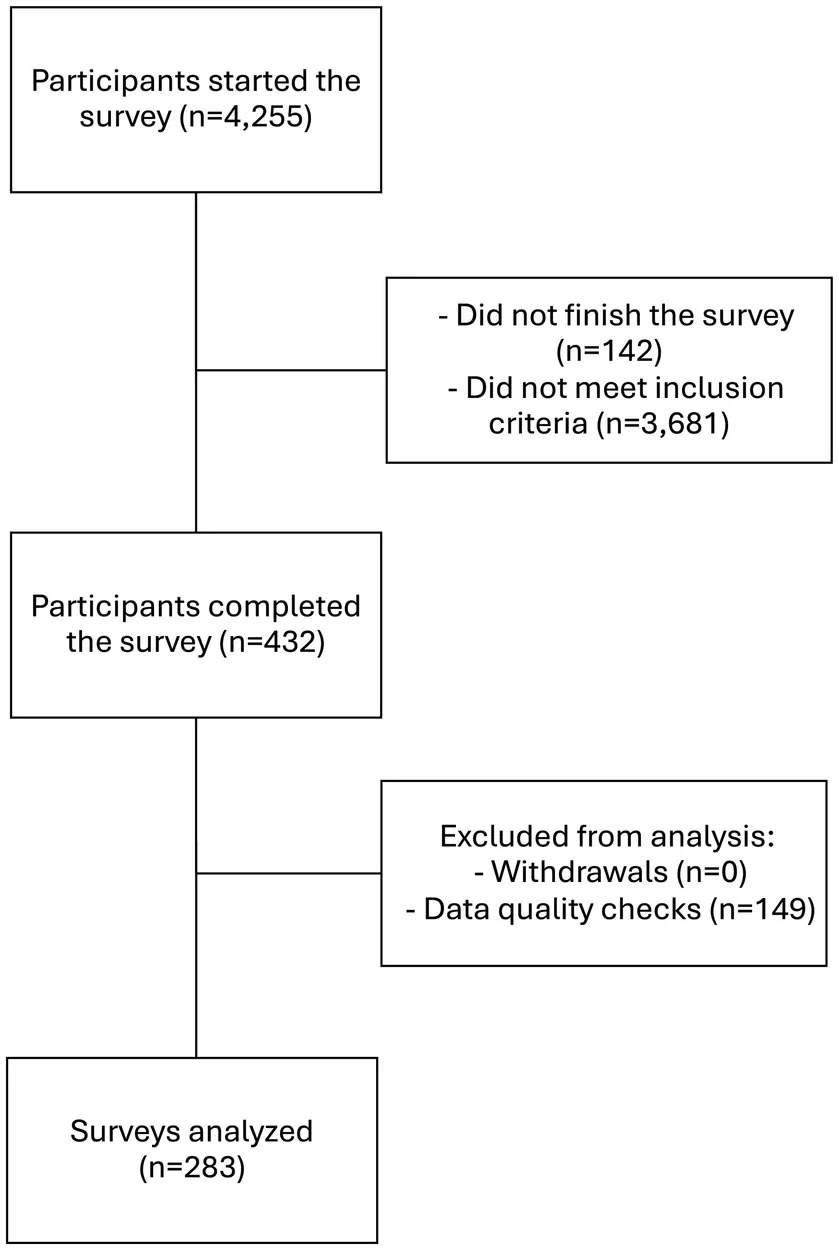
Flowchart of respondents.

**Table 1:** Patient demographic and medical characteristics.

Characteristics	Patient respondent (*n* = 221)	Caregiver respondent (*n* = 62)	All patients (*n* = 283)
Age at first stroke, median (IQR), y	45.5 (37.0–56.1)	44.6 (34.5–61.4)	45.4 (36.7–57.2)
Time since first stroke, median (IQR), mo	10.8 (6.0–26.4)	13.2 (6.0–45.6)	10.8 (6.0–31.2)
Type of the most recent stroke in the past year, *n* (%)			
First stroke event	130 (58.8)	30 (48.4)	160 (56.5)
Repeat stroke event	91 (41.2)	32 (51.6)	123 (43.5)
Number of strokes, median (IQR)	1.0 (1.0–2.0)	2.0 (1.0–2.8)	2.0 (1.0–2.0)
Most recent type of stroke, *n* (%)			
Ischemic	167 (75.6)	49 (79.0)	216 (76.3)
Hemorrhagic	34 (15.4)	9 (14.5)	43 (15.2)
Do not know/do not remember	20 (9.0)	4 (6.5)	24 (8.5)
Comorbidities, *n* (%)			
Cardiovascular diseases	103 (46.6)	26 (41.9)	129 (45.6)
Metabolic diseases	82 (37.1)	30 (48.4)	112 (39.6)
Pulmonary diseases	66 (29.9)	12 (19.4)	78 (27.6)
Autoimmune diseases	32 (14.5)	7 (11.3)	39 (13.8)
Neurologic diseases (other than stroke)	70 (31.7)	26 (41.9)	96 (33.9)
Depression	62 (28.1)	23 (37.1)	85 (30.0)
Other conditions	16 (7.2)	2 (3.2)	18 (6.4)

IQR, interquartile range.

### Patient experiences with stroke and spasticity

#### Symptoms

Almost all respondents (98%, 277/283) indicated that patients experienced PSS symptoms after their most recent stroke, and more than half (59%, 166/283) indicated patients experienced ≥3 symptoms. The most frequently reported symptoms were difficulties moving the leg (57%, 161/283), muscle pain (54%, 154/283), and difficulties moving the arm and/or hand (54%, 152/283). Significant differences were observed between the caregiver group and the patient group: caregivers more frequently reported patients having difficulties moving the arm/hand (74%, 46/62) compared to the patient group (48%, 106/221; *p* < 0.001). Caregivers also reported a higher mean (SD) number of patient symptoms than did patients themselves [3.5 (1.5) vs. 2.9 (1.6); *p* = 0.007], and more frequently reported patients having difficulties moving their leg (73%, 45/62 vs. 53%, 116/221; *p* = 0.007).

Half of the respondents who experienced or reported PSS symptoms (50%, 139/277) started noticing them in the past 6 months. In most cases (74%, 204/277), patients were the first to notice PSS symptoms. Symptoms were also noticed by a third party for 54% (148/277) of the respondents. In a large majority of cases (94%, 259/277) patients had their PSS symptoms managed by a healthcare professional. For most (61%, 169/277), PSS management started within the first week after onset.

When questioned on the level of information received on different topics related to PSS (how to prevent PSS, PSS treatments, medical examination to diagnose PSS, risk of PSS associated with stroke, symptoms associated with PSS), 43 to 50% of the patients reported wishing they had received more information. The topic for which patients reported the greatest need for more information was how to prevent PSS (50%, 142/283).

#### Treatments

In the majority of cases (91%, 257/283), patients received ≥1 form of rehabilitation therapy following their most recent stroke. Physical therapy was the most frequently reported (81%, 229/283), followed by occupational therapy (48%, 136/283) and speech therapy (43%, 121/283). Most patients (57%, 161/283) received ≥2 types of therapy.

Among the patients suffering from PSS symptoms, 56% (155/277) were aware of the availability of drug treatments. Of treatment-aware respondents, 95% (147/155) had received ≥1 medication for their PSS symptoms. Most (88%, 134/155) received ≥1 oral medication, 60% (93/155) received intramuscular injections, and 41% (64/155) reported receiving intrathecal baclofen therapy either currently or in the past.

Among patients with PSS symptoms, male patients or their caregivers were more likely to report being aware of drug treatment availability for managing symptoms (64%, 98/153) compared to their female counterparts or their caregivers (46%, 57/124, *p =* 0.004). Female patients were less likely to have received intramuscular injections (42%, 24/57) than male patients (70%, 69/98, *p =* 0.002). Intrathecal baclofen therapy was received by 52% of male patients (51/98) and only 23% of female patients (13/57, *p =* 0.001).

Among the 147 patients who received ≥1 medication for PSS symptoms, 68% (*n* = 100) were involved in the initial drug selection, either by requesting a specific treatment (31%, 45/147) or by choosing among options offered by their healthcare professional (37%, 55/147; Figure 2). Most (79%, 79/100) wished to have been even more involved in the selection of their first treatment. Male patients were more likely to report having been involved in the initial drug selection [72% (68/95)], with 40% (38/95) requesting a treatment and 32% (30/95) choosing between several options, compared to 62% (32/52) of female patients, of whom 14% (7/52) requested a treatment and 48% (25/52) chose between options (*p =* 0.004). Younger patients (<48 y) were also more likely to have been involved in the choice of their treatment (81%, 67/83) than older patients (52%, 33/64, *p <* 0.001).

**Figure 2:**
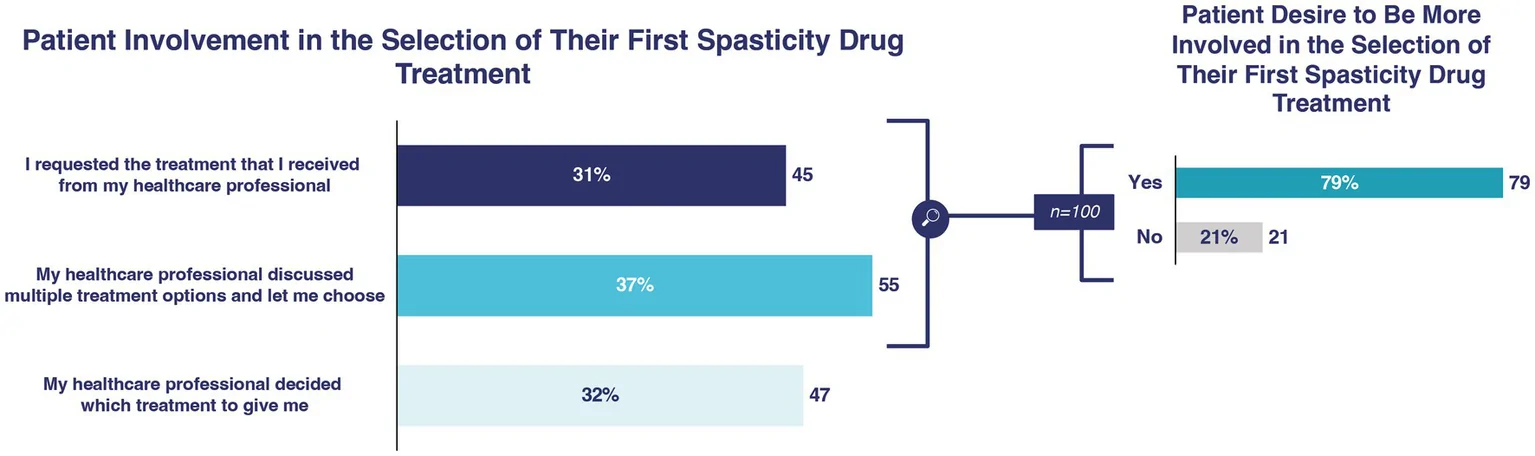
Patient involvement in the selection of their first PSS drug treatment (*n* = 147^a^). ^a^Patients who took ≥1 drug treatment for PSS symptoms. PSS, poststroke spasticity.

### Impact of stroke and spasticity on QoL

Over half of patients or their caregivers reported difficulties in patients carrying out their daily activities (Figure 3). The most commonly affected tasks were household chores, with 82% (232/283) experiencing at least some difficulty. This was followed by walking or using a wheelchair without resting (74%, 209/283) and needing assistance with bathing or showering (64%, 181/283).

**Figure 3:**
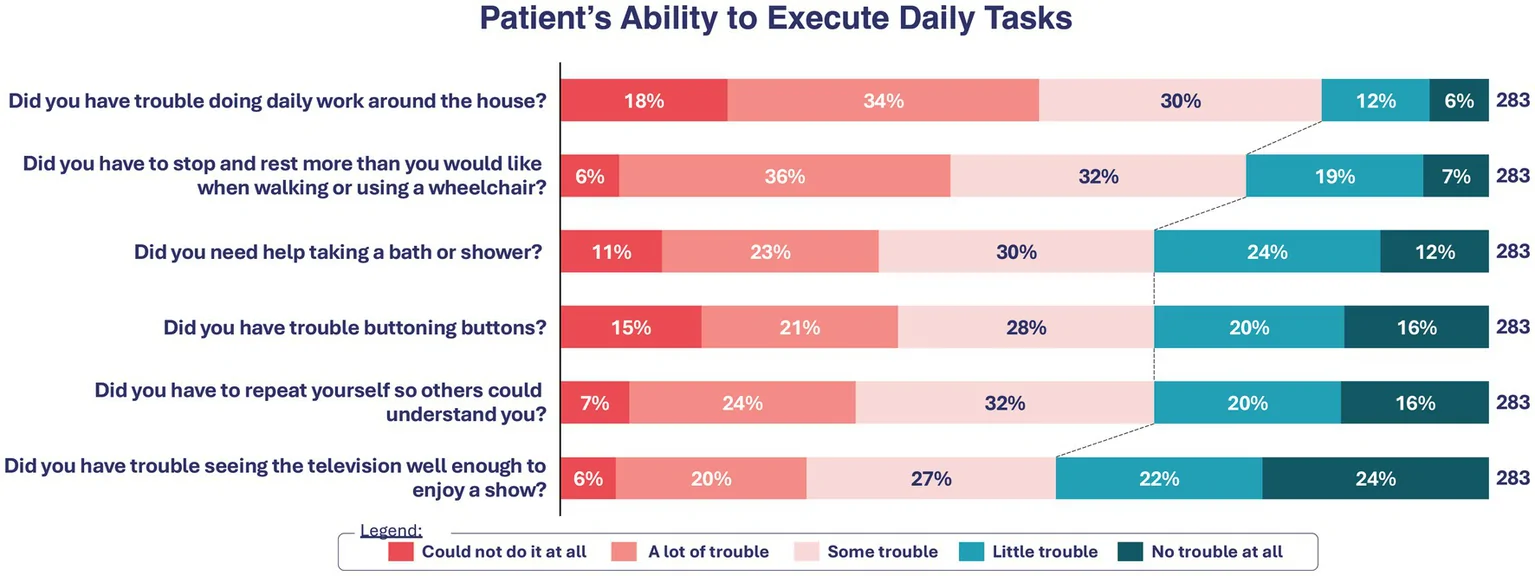
Patients’ ability to execute daily tasks (*n* = 283).

The caregiver groups reported significantly more troubles with showering or bathing unassisted (84%, 52/62) and walking/using a wheelchair without stopping (90%, 56/62) compared to the patient group (58%, 129/221, *p* < 0.001, for bathing or showering; 69%,153/221, *p* = 0.002, for walking or using a wheelchair).

Beyond their ability to perform daily tasks, patients also reported a considerable impact of stroke on several other aspects of their life (Figure 4). The most impacted aspect were fatigue, social life, and future life: out of the 283 patients, 237 (84%) reported feeling too tired to do what they wanted to, 81% (231/283) agreed that their condition interfered with their social life, and 75% (212/283) reported feeling discouraged about their future.

**Figure 4:**
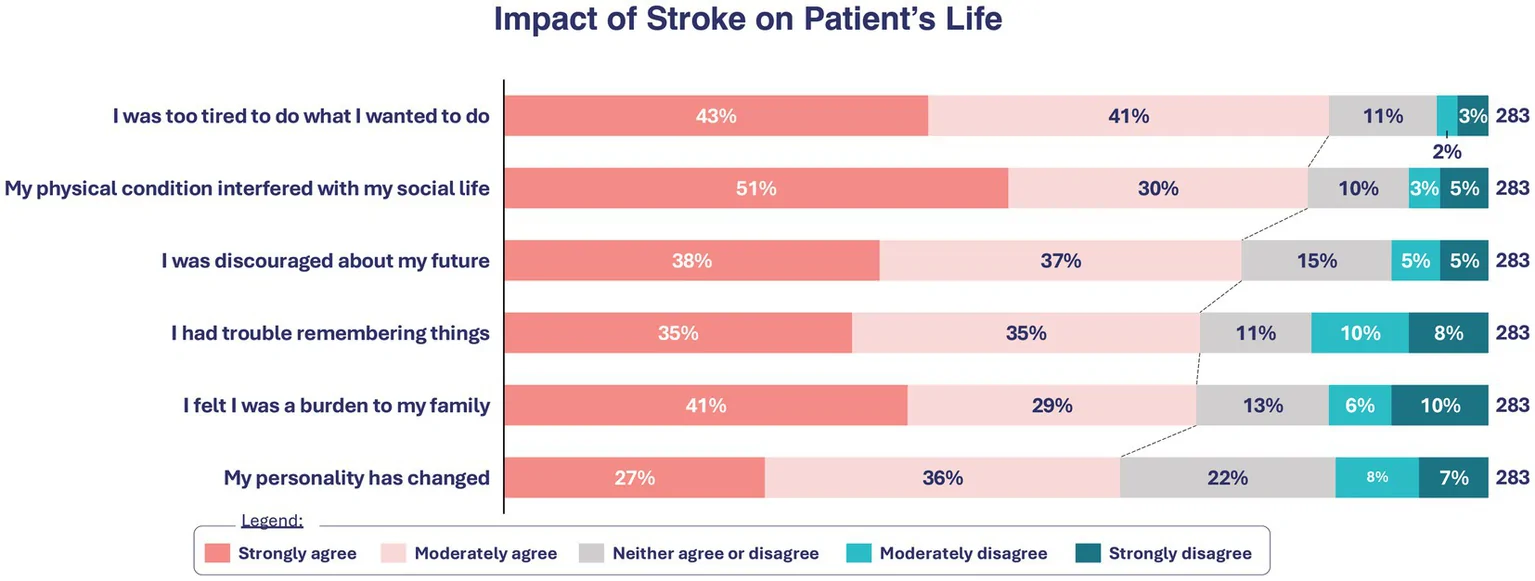
Reported impact of stroke on different aspects of patient life (*n* = 283).

Younger respondents (<48 y) were more likely to feel like a burden to their family (80%, 110/137) and to worry about personality changes (73%, 100/137) than older patients (61%, 89/146; *p* = 0.008 and 53%, 77/146; *p* = 0.012, respectively). The overall median SS-QoL score was 2.5 (IQR: 2.0–2.9; min, max: 1, 4.9). The median SS-QoL score was higher in the patient group (2.5, IQR: 2.1–2.9) than for patients from the caregiver group (2.1, IQR: 1.8–2.8, *p* = 0.001), as well as in younger patients (2.4, IQR: 1.8–2.8) compared to older ones (2.6, IQR: 2.2–3.0, *p* = 0.002, Table 2). Median values were similar across sex and type of strokes (ischemic or hemorrhagic) groups. The multivariate linear regression model performed on the subgroup of patients whose most recent stroke in the past year was a first stroke (*n* = 160) assessed the impact of the patient profile, comorbidities, symptoms, treatments, and therapies on the SS-QoL score. The SS-QoL score was significantly (*p* < 0.001) and independently associated with receiving speech therapy and having difficulties moving the arm and/or hand, suggesting a lower QoL in individuals with greater functional and communication impairments (Table 3).

**Table 2:** Median SS-QoL score by profile, sex, age group, and type of last stroke.

	*n*	Median SS-QoL score (IQR)	*p* value
Profile			
Patient group	221	2.5 (2.1–2.9)	0.001
Caregiver group	62	2.1 (1.8–2.8)	
Sex			
Men	155	2.5 (2.0–2.8)	0.599
Women	128	2.5 (2.0–2.9)	
Age			
<48 y	137	2.4 (1.8–2.8)	0.002
≥48 y	146	2.6 (2.2–3.0)	
Type of last stroke			
Ischemic	216	2.5 (1.9–2.9)	0.077
Hemorrhagic	43	2.4 (2.1–2.8)	
Do not know/ do not remember	24	2.8 (2.4–3.3)	

IQR, interquartile range; SS-QoL, Stroke-Specific Quality of Life Scale.

**Table 3:** Results from the reduced multivariate linear regression model for factors predicting adapted SS-QoL mean score in patients whose most recent stroke <1 year prior was a first stroke (*n* = 160).

Variables	*n*	*β*	95% CI	*p* value
Speech therapy				
No	88	Reference		
Yes	72	−0.43	−0.65, −0.22	<0.001
Difficulties moving the arm/hand				
No	65	Reference		
Yes	95	−0.40	−0.62, −0.19	<0.001

SS-QoL, Stroke-Specific Quality of Life Scale.

## Discussion

To better understand the impact of PSS on QoL, this cross-sectional, web-based survey collected real-world data from respondents who had experienced a stroke with hemiparesis within the past year. Nearly all patients reported symptoms of spasticity following their most recent stroke with hemiparesis, thereby confirming the well-established correlation between poststroke hemiparesis and spasticity (8). Previous studies have typically reported a lower prevalence of spasticity in patients with paresis, ranging from 27 to 49% within 12 months of follow-up (23–25, 28–30). This discrepancy in spasticity prevalence may be explained by differences in patient populations and study designs, including a selection bias inherent to self-reported surveys, with a tendency toward greater participation from individuals experiencing persistent or severe motor symptoms, as well as the possibility of self-report bias if survey participants were to misclassify related symptoms such as cramps, contractures, pain, or paresis as PSS. Regardless, this finding highlights the substantial burden of spasticity perceived by patients and caregivers in real-world settings.

Our study provides valuable insights into the awareness and knowledge of spasticity among patients and their caregivers. Interestingly, almost half of the patients expressed a wish for more information about spasticity, mainly because they wanted to know how to prevent it. Approximately half of the respondents were aware of pharmacologic options for spasticity, and most of them wished to be more involved in treatment decisions. These findings further demonstrate the limited awareness of spasticity and its treatments, highlighting the need to strengthen patient education and promote shared decision-making in spasticity management. This outcome is consistent with the published evidence of inadequate knowledge regarding spasticity symptoms and treatment benefits among healthcare providers, patients, and caregivers, as well as limited access to specialized care and inadequate patient follow-up (25). Furthermore, the results of the ongoing EPITOME study, evaluating a remote monitoring system for the detection of PSS in stroke survivors, may complement and further inform on the true rates of PSS and interventions (31).

In this study, we also observed that PSS exerts a considerable impact on QoL, largely driven by fatigue and limitations in daily activities. Indeed, 84% of patients reported feeling too tired to do what they wanted. This finding is consistent with prior research demonstrating that PSS alters functional abilities, mobility, and activities of daily living (7, 28–30). The presence of a restricted range of motion, muscle stiffness or contractures, pain, and lack of vitality have also been documented in patients with spasticity (29, 30). Altogether, these findings reinforce the pivotal role of fatigue and functional limitations in mediating the detrimental effect of PSS on QoL, which may be particularly relevant in younger stroke survivors.

Importantly, several subgroup comparisons revealed significant differences that warrant further attention. Patients and caregivers reported different perceptions of PSS symptoms and their impact; for instance, caregivers more frequently reported functional difficulties, such as problems with arm/hand mobility and bathing, and also rated QoL lower than did the patients themselves. These findings could suggest a possible underestimation of functional limitations by patients or a heightened awareness of disease burden among caregivers, which may reflect their close involvement in daily care and observation. However, this could also suggest additional baseline differences between patients who answered the survey themselves and those whose caregiver responded, though this was not specifically assessed in this study. This divergence in perception emphasizes the importance of integrating both patient and caregiver perspectives into treatment planning and evaluation, and warrants further study to determine whether differences in perception reflect patient characteristics or genuine patient-caregiver reporting differences.

Moreover, additional subgroup analyses revealed that sex and age influenced awareness and engagement in treatment. Male and younger patients were more likely to report being involved in treatment choices and more aware of pharmacologic options, whereas female patients reported lower involvement and lower use of advanced treatment modalities, such as intramuscular injections or intrathecal therapy. These discrepancies suggest a possible need to tailor educational and support interventions based on patient demographics to avoid disparities in care.

Most respondents (81%) also reported a considerable impact of PSS on their social life, which is consistent with the results of Facciorusso et al. (28), who showed that spasticity was associated with psychosocial issues in terms of mood, sleep quality, relationships, and social life. Similarly, a web-based survey by Ganapathy et al. (19) showed that while PSS profoundly affects the QoL of patients, the psychologic burden of PSS-related disability often weighs even more heavily on caregivers than on stroke survivors (18). Caregiving has also been shown to be associated with a loss of productivity because of work restrictions, absenteeism, presenteeism, and indirect costs related to patient care, thereby further aggravating the disease burden (18).

It is worth noting that this study presents several strengths and limitations that should be considered when interpreting the data. A key strength of the study lies in the collection of real-world data directly from patients and their caregivers, providing valuable insights into their lived experiences, unmet needs, and treatment gaps in current clinical practice. With a sample size of 283 respondents, the study offers a meaningful overview of stroke management and QoL across this patient population, and is larger than other recent surveys of patients with PSS (32, 33). The use of the validated SS-QoL-12, which has been shown to capture 88 to 95% of the variance of the original 49-item scale (27), allowed for efficient assessment of QoL while minimizing respondent burden. The self-administered online format enabled participants to complete the survey at their own pace and express themselves directly, thus minimizing the potential for bias in social desirability. However, several limitations must be acknowledged, including the susceptibility to biases such as self-report bias, recall bias, social desirability bias, and selection bias due to recruitment through online communities and advocacy organizations, which may have attracted individuals with more persistent or severe symptoms, greater functional impact, or more intensive treatment needs. Several measures were implemented to reduce potential sources of bias. In the absence of clinical diagnosis, the survey’s symptom questions were specifically designed around hallmark PSS symptoms, to mitigate self-report bias and minimize symptom misclassification. Recruitment through multiple channels (the Carenity community, partner panels, and online advertising campaigns) was intended to broaden participation and reduce selection bias. To reduce recall bias, patients had to have experienced a stroke within the past year; and participants could pause and resume the questionnaire to retrieve missing information. The study also relied on validated patient-reported outcome instruments where available, carefully designed questionnaire wording, and data quality checks to ensure the consistency of health-related information. Finally, to reduce the social desirability bias, no interviewers or healthcare professionals were involved; the administration of the survey was online and pre-specified answer options were provided for most questions. The reliance on self-reported data may also have introduced variability in responses, particularly regarding subjective symptoms, treatments, and treatment adherence. Because reports were not clinically verified, we cannot exclude the possibility of appreciable misunderstanding or misclassification by respondents, particularly regarding advanced treatment modalities such as intrathecal baclofen therapy, which was not explicitly explained in the survey question about treatments used (see Appendix). Additionally, the online survey design limits the ability to control for potential confounding factors, such as differences in patient demographics and disease characteristics. The relatively young age distribution of the study sample may have skewed the results; our sample was collected via a web-based community, making it likely that respondents would be younger. The absence of broader demographic data further limits the representativeness and generalizability of the study population. Furthermore, it was possible for both a patient and their caregiver to participate independently in the study, as no patient–caregiver pairing was implemented. While significant subgroup differences were observed, these findings should be interpreted with caution as exploratory only, owing to the relatively small sample sizes of certain subgroups, the lack of pre-testing for statistical power, and the lack of multiplicity adjustment applied. Additionally, no power calculations or hypotheses were predefined to adjust for population differences. Further studies are needed to confirm these trends, determine their clinical relevance, and understand both the differences in patient–caregiver perceptions of PSS impact on QoL disease and treatment knowledge between male and female patients.

In conclusion, this study provides valuable real-world insights into the demographic profiles, symptoms, and treatment of patients with PSS, as well as the significant QoL deterioration associated with this condition. Notably, it highlights differences in perceptions and experiences between patients and caregivers, reinforcing the importance of a double approach in assessing and addressing the burden of PSS. Beyond the optimization of stroke management and the alleviation of symptoms, our findings highlight the need to prioritize the improvement of QoL and the mitigation of caregiver burden. Targeted interventions that consider age, sex, and caregiver involvement are critical to ensure equitable access to care and support. Multidisciplinary interventions are essential to enhance patient outcomes and support caregivers. Finally, raising awareness of spasticity among healthcare providers, patients, and caregivers is critical to promote timely diagnosis and adequate management of PSS.

## Data Availability

The raw data supporting the conclusions of this article will be made available by the authors, without undue reservation.
