# Supplementary material for: Understanding stroke and spasticity: patient and caregiver experiences and quality-of-life impact
Source: Front Neurol. 2026 Oct 15;17:1849865. doi: 10.3389/fneur.2026.1849865 (PMC13718017; doi:10.3389/fneur.2026.1849865)
Supplement: Supplementary file 1 [file Table_1.DOCX]

**Introductory message for Carenity members:**

Hello,

We would like to invite you to participate in a survey dedicated to adult patients who have had a stroke and their caregivers.

The aim of this study is to better understand the knowledge and experience of patients. This survey is being conducted with adult patients or their caregivers in the US. The survey is scheduled to last no longer than 20 minutes.

The data collected through this study will be shared anonymously and in aggregate form, which means that it will not be possible to identify the participants.

These aggregate results will be shared with Ipsen, a pharmaceutical company developing treatments for post-stroke patients.

The aggregate results of this study may also be used for external communications (in aggregated form).

*According to current legislation, you have the right to access, modify and oppose to the processing of your data at any time.*

*To learn more about the way your data will be used and about the terms of your rights, please read the Information and Consent Notice.*

*Your participation in the survey is voluntary. By clicking on the “Start” button, you confirm:*

*1. your willingness to participate in the study; and*

*2. that you give your explicit consent to have your personal data, including sensitive data (e.g. health information) collected and processed in the way described in the Information and Consent Notice.*

There are no right or wrong answers, and you are not obliged to answer any questions you do not want to.

You are free to leave the survey, or study, at any time. Your participation is entirely voluntary. There is no payment for completion, but your support is much appreciated.

Thank you in advance for your participation,

The Carenity Team

A- **SCREENER**

**Message to be displayed to participants: The first questions will allow us to ensure you meet the inclusion criteria.**

1. **Your profile:**

*Please select only one answer.*

- Patient who has had a stroke
- Caregiver of a patient who has had a stroke
- None of the above **[END SURVEY]**

**Message to be displayed to caregivers: Please answer to all questions for the patient and not for yourself even when you feel the question is addressed for you.**

2. **Your year of birth:**

*Please select a year in the calendar by clicking the field below.*

(YYYY)

**[IF THE PATIENT IS AGED YOUNGER THAN 18 YEARS OLD, END SURVEY]**

3. **Your country of residence:**

*Please select only one answer.*

- United States of America
- Other **[END SURVEY]**

4. **When did your most recent stroke occur?**

*If you do not remember the exact date, please give your best estimate.*

(MM/YYYY)

**[IF THE PARTICIPANT HAD THEIR LATEST STROKE MORE THAN 12 MONTHS AGO, END SURVEY]**

5. **How many strokes have you had?**

*Numerical field, range 1-10.*

**Q6 will be displayed to participants who have had more than 1 stroke.**

6. **When did your first stroke occur?**

*If you do not remember the exact date, please give your best estimate.*

(MM/YYYY)

7. **Did you experience any weakness in your arm and/or leg after your most recent stroke?**

*Please select only one answer.*

- Yes
- No **[END SURVEY]**

B- **SOCIODEMOGRAPHIC AND MEDICAL PROFILE**

**Message to be displayed to participants: Thank you for starting our survey! The following questions will focus on your sociodemographic and medical profile.**

8. **Your gender:**

*Please select only one answer.*

- Male
- Female
- Other
- Prefer not to answer

9. **Which of the following medical conditions are you currently living with?**

*Please select all that apply.*

*A medical condition is a broad term that includes all diseases, lesions, and disorders.*

- Cardiovascular diseases (e.g., arterial hypertension, heart attack, heart failure)
- Metabolic diseases (e.g., diabetes, obesity)
- Pulmonary diseases (e.g., Chronic Obstructive Pulmonary Disease (COPD), asthma, pneumonia, pulmonary embolism)
- Auto-immune diseases (e.g., lupus, primary biliary cirrhosis, autoimmune hepatitis)
- Neurological diseases (**other than stroke**, e.g., epilepsy, chronic migraine)
- Depression
- Other
- None **[EXCLUSIVE]**

C- **PATIENT JOURNEY WITH STROKE AND SPASTICITY**

**Message to be displayed to participants who had several strokes: For the rest of the questionnaire, we will only focus on your most recent stroke.**

10. **What was your most recent stroke type?**

*Please select only one answer.*

- An ischemic stroke (stroke caused by blockage/clot of an artery or, in rare instances, a vein)
- A hemorrhagic stroke (stroke caused by bleeding)
- I do not know/ I do not remember

11. **Following your most recent stroke, what type of therapy did you receive?**

*Please select all that apply.*

- Physical therapy
- Occupational therapy
- Speech therapy
- Other
- None **[EXCLUSIVE]**
- I do not know/ I do not remember **[EXCLUSIVE]**

12. **Which of the following symptoms have you experienced following your most recent stroke?**

*Please select all that apply.*

- Muscle spasms
- Muscle stiffness/rigidity (including painful cramps)
- Muscle pain
- Unwanted movement of the affected arm and/or leg
- Difficulties moving my leg, which may result in falling, tripping, loss of balance
- Difficulties moving my arm/hand, extending my arm, and/or opening my hand
- None of the above **[EXCLUSIVE]**

**Message to be displayed to participants who have indicated spasticity in Q12: In the following questions, we will use the word “spasticity” to refer to the symptoms you selected in the previous question among muscle spasms, muscle stiffness/rigidity, muscle pain, unwanted movement of the affected limb, difficulties using the leg (i.e. falling, tripping, loss of balance), or difficulties using the arm/hand (extending the arm, opening the hand…).**

**Q13 to Q20 will only be displayed to participants who have indicated spasticity in Q12.**

13. **When did you start experiencing your spasticity symptoms following your most recent stroke?**

*If you do not remember the exact date, please give your best estimate.*

(MM/YYYY)

14. **Who first noticed your spasticity symptoms following your most recent stroke?**

*Please select all that apply.*

- Me
- A friend or relative
- A healthcare professional (doctor, physical therapist, occupational therapist, nurse,etc.)
- Other
- I do not remember **[EXCLUSIVE]**

15. **Following your most recent stroke, how long was the time between when your first spasticity symptoms started and their management by a healthcare professional?**

*Please select only one answer.*

- A day
- A week
- A month
- Several months
- I do not remember
- My spasticity symptoms are not managed by a healthcare professional

**Q16 will only be displayed to participants who have not indicated “My spasticity symptoms are not managed by a healthcare professional” in Q15.**

16. **Which of the following treatment methods have you received for your spasticity symptoms following your most recent stroke?**

*Please select all that apply.*

- Physical or occupational therapy
- Bracing/casting/splints
- Surgery
- Other
- None **[EXCLUSIVE]**

17. **Were you aware that drug treatments were available for your spasticity symptoms?**

*Please select only one answer.*

- Yes
- No

**Q18 will only be displayed to participants who have indicated knowing treatments for spasticity symptoms in Q17.**

18. **Which treatment do you take for your spasticity symptoms?**

*One answer per line.*

|  | I am currently receiving this treatment and it is my first treatment | I am currently receiving this treatment but it is not my first treatment | I have taken this treatment in the past and it was my first treatment | I have taken this treatment in the past and it was not my first treatment | I have never taken this treatment |
| --- | --- | --- | --- | --- | --- |
| Oral medication (for instance, muscle relaxant, oral baclofen) | ○ | ○ | ○ | ○ | ○ |
| Injections into the muscle (for instance, Botulinum Toxin A  injections like Botox, Dysport, Xeomin) | ○ | ○ | ○ | ○ | ○ |
| Intrathecal baclofen therapy | ○ | ○ | ○ | ○ | ○ |
| Other | ○ | ○ | ○ | ○ | ○ |

**Q19 will only be displayed to participants who have indicated having taken at least one treatment in Q18.**

19. **Which of the following describes your involvement in the selection of your first spasticity drug treatment(s)?**

*Please select the best answer.*

- I requested the treatment that I received from my healthcare professional
- My healthcare professional discussed multiple treatment options and let me choose
- My healthcare professional decided which treatment to give me

**Q20 will only be displayed to participants who have not indicated “My healthcare professional decided which treatment to give me” in Q19.**

20. **Would you have liked to have been more involved in the selection of your first spasticity drug treatment(s)?**

*Please select only one answer.*

- Yes
- No

D- **IMPACT OF STROKE ON PATIENTS’ QUALITY OF LIFE**
21. **To what extent did you experience trouble in the following aspects?**

*One answer per line.*

|  | Could not do it at all | A lot of trouble | Some trouble | Little trouble | No trouble at all |
| --- | --- | --- | --- | --- | --- |
| Did you need help taking a bath or shower? | ○ | ○ | ○ | ○ | ○ |
| Did you have to stop and rest more than you would like  when walking or using a wheelchair? | ○ | ○ | ○ | ○ | ○ |
| Did you have trouble buttoning buttons? | ○ | ○ | ○ | ○ | ○ |
| Did you have to repeat yourself so others could  understand you? | ○ | ○ | ○ | ○ | ○ |
| Did you have trouble seeing the television well enough to  enjoy a show? | ○ | ○ | ○ | ○ | ○ |
| Did you have trouble doing daily work around the house? | ○ | ○ | ○ | ○ | ○ |

22. **To what extent do you agree with the following aspects?**

*One answer per line.*

|  | Strongly agree | Moderately agree | Neither agree or disagree | Moderately disagree | Strongly disagree |
| --- | --- | --- | --- | --- | --- |
| I had trouble remembering things | ○ | ○ | ○ | ○ | ○ |
| I felt I was a burden to my family | ○ | ○ | ○ | ○ | ○ |
| My physical condition interfered with my social life | ○ | ○ | ○ | ○ | ○ |
| My personality has changed | ○ | ○ | ○ | ○ | ○ |
| I was discouraged about my future | ○ | ○ | ○ | ○ | ○ |
| I was too tired to do what I wanted to do | ○ | ○ | ○ | ○ | ○ |

23. **Did your healthcare team in the hospital provide you any of the following information?**

*One answer per line.*

|  | Yes, and I received all the information I needed | Yes, but I would have liked to receive more information | Yes, but I did not need as much information | No, but I would have liked to receive information | No, but it was not necessary |
| --- | --- | --- | --- | --- | --- |
| Information on risk of spasticity associated to stroke | ○ | ○ | ○ | ○ | ○ |
| Information on symptoms associated to spasticity | ○ | ○ | ○ | ○ | ○ |
| Information on medical examination to diagnose spasticity | ○ | ○ | ○ | ○ | ○ |
| Information on how to prevent spasticity | ○ | ○ | ○ | ○ | ○ |
| Information on spasticity treatment | ○ | ○ | ○ | ○ | ○ |
